# Effect of nutrition behaviour change communication delivered through radio on mothers’ nutritional knowledge, child feeding practices and growth

**DOI:** 10.1017/jns.2021.35

**Published:** 2021-06-07

**Authors:** Mahama Saaka, Khadija Wemah, Fred Kizito, Irmgard Hoeschle-Zeledon

**Affiliations:** 1 School of Allied Health Sciences, University for Development Studies, P.O. Box 1883, Tamale, Ghana; 2 Ghana Health Service, Savelugu Nanton District Health Directorate, P.O. Box 45, Savelugu, Ghana; 3 International Institute of Tropical Agriculture (IITA), P.O. Box 6, Tamale, Ghana; 4 International Institute of Tropical Agriculture (IITA), PMB 5320, Oyo Road, Ibadan, Nigeria

**Keywords:** Minimum dietary diversity, Mothers’ nutritional knowledge, Northern Ghana, Nutrition education on radio, Nutrition-related attitudes, CF, complementary feeding, DID, difference-in-difference, IYCF, infant and young child feeding, KAP, knowledge, attitudes and practices, LAZ, length-for-age, MM, mass media, MAD, minimum acceptable diet, MDD, minimum dietary diversity, MMF, minimum meal frequency, NKS, nutrition knowledge score, WAZ, weight-for-age, WLZ, weight-for-length

## Abstract

Childhood undernutrition coupled with poor feeding practices continues to be public health problems in many parts of the world and efforts to address them remain elusive. We tested the hypothesis that women who are exposed to radio health/nutrition education will demonstrate greater nutrition and health knowledge, positive attitudes towards preventive health and better dietary diversity practices for improved child growth. We used a two-arm, quasi-experimental, non-equivalent comparison group design with pre- and post-test observations to evaluate the intervention. The study population comprised 712 mothers with children aged 6–36 months who were randomly selected from five intervention districts and one comparison district in Northern Ghana. Difference-in-difference (DID) analysis was performed to assess study outcomes. After 12-month implementation of intervention activities, the minimum dietary diversity and the minimum acceptable diet improved significantly (DID 9⋅7 percentage points, *P* 0⋅014 and DID 12⋅1 percentage points, *P* 0⋅001, respectively) in the intervention study group, compared with the comparison group. Mothers in the intervention communities had a nutrition-related knowledge, attitudes and practices score that was significantly higher than their colleagues in the comparison communities (DID 0⋅646, *P* < 0⋅001). The intervention did not have significant effects on the nutritional status as measured by height-for-age *Z*-score or weight-for-height *Z*-score. The data provide evidence that health and nutrition education using radio drama significantly increased health-/nutrition-related knowledge but had little effect on nutritional status.

## Introduction

Childhood undernutrition remains a global public health problem that affects many middle-income countries, including Ghana, as many as 165 million children are estimated to be stunted and it is an underlying cause of 3⋅1 million child deaths annually^(1)^. In 2016, 45 % of the 5⋅6 million children under the age of 5 years who died were attributed to undernutrition^(2)^. The problem continues to evade world efforts at its eradication, so potential interventions and strategies are being sought for a lasting solution. One such intervention is the promotion of appropriate infant and young child feeding (IYCF) through behaviour change communication. This approach is derived from the fact that having sound nutrition knowledge is an essential facilitating factor for addressing poor diets and inadequate food intake. The most important information for mothers is the kinds of food to eat and how to prepare the food in the right quantities and mixes and in a way that is safe and clean for children's healthy growth and development. Behaviour change interventions are key to providing appropriate information for mothers that could change their feeding behaviours^(3)^. Therefore, empowering women, especially mothers with nutrition knowledge and skills, has a greater potential to impact positively on child nutritional outcomes. Nutrition education generally seeks to increase nutritional knowledge, thereby influencing attitude and practices towards good nutrition^(4,5)^. Nutritional knowledge and attitude are important factors of dietary practices, so mother's knowledge of nutrition is critical for good pregnancy outcomes and improving children's nutritional status^(6)^. Furthermore, the need to deliver nutrition education to a large segment of the population using effective and efficient strategies cannot be over-emphasised. Mass media (MM), including radio, is one way that this can be achieved but for which evidence is lacking.

In Northern Ghana, complementary feeding (CF) practices are extremely poor^(7,8)^, and efforts to address the problem remain elusive. Effective interventions and strategies are urgently needed for a lasting solution. It is against this background that the International Institute of Tropical Agriculture (IITA), Tamale supported the Department of Nutritional Sciences of the University for Development Studies and Ghana Health Service to embark on public nutrition education on radio to raise awareness about nutrition issues, including child feeding in Northern Ghana, where stunted growth is widespread^(7,9)^.

This current intervention study, therefore, tested the hypothesis that mothers/caretakers who are exposed to radio health/nutrition education will demonstrate improved nutritional knowledge of mothers, positive attitudes towards preventive health and better dietary diversity practices for improved child growth.

## Materials and methods

### Evaluation design

A two-arm, quasi-experimental, non-equivalent comparison group design with pre- and post-test observations was used to quantify the effectiveness of a radio listening behaviour. This study was conducted according to the guidelines laid down in the Declaration of Helsinki, and all procedures involving human subjects/patients were approved by the Ethics Committee of the Navrongo Health Research Center (Reference No. NHRGIRB353). Written informed consent was obtained from all subjects/patients.

### Study population, sample size and sampling

The study population comprised mothers with children aged 6–36 months who were randomly selected from five intervention districts (*n* 412) and one comparison district (*n* 300). The sample was estimated based on the primary outcome of child linear growth, which yielded the maximum sample size^(10)^. The sample size was thus calculated providing a statistical power of 80 % at 95 % confidence level to detect a reduction of 10-percentage point difference in the prevalence of stunting between the study groups. The sample size was estimated using OpenEpi software for epidemiologic statistics version 3.01.

In each selected cluster, a sample size of sixteen households with eligible mothers/caretakers and children was systematically selected. A complete list of all households was compiled, and systematic random sampling was used in selecting the households. All the households in each cluster were serially numbered. To get the sampling interval, the total number of households in a cluster was divided by the sample size of 16. The first household was then randomly selected by picking any number within the sample interval. Subsequent selections were made by adding the sampling interval to the selected number in order to locate the next household to visit. If the selected household does not have a target respondent, then the next household was selected using the systematic sampling procedure. This process continued until the required sample size was obtained. Only one eligible participant was selected from each household for household interview, using simple random sampling.

### Description of the nutrition education intervention

This radio health/nutrition education was jointly implemented by the Department of Nutritional Sciences of the University for Development Studies (UDS) and the District Health Directorates of Savelugu, Kassena/Nankana and Wa West. The rationale behind the programme was to maximise the reach of nutrition-related messages through radio waves to targeted audience. The intervention consisted of a series of recorded health and nutrition drama that were broadcast for a 12-month duration in local dialects on five radio stations (Radio Upper West, Nabiina Community Radio, Zaa Radio, Radio Wa and Radio Justice).

The dramas were produced in local languages spoken by a team of radio actors and scriptwriters. The intervention in the form of providing nutrition education once in a week had been conducted for 12 months. The radio material used was recorded drama series whose content included nutrition for pregnant women and lactating mothers, appropriate CF practices, planning diversified diets for the household, active feeding, the importance of consumption of fruits and vegetables for health and different ways men can help their wives to improve nutrition in the family.

The drama series were aired once a week usually at the night (20 h through 22 h). The listening public was invited to phone-in to ask questions and clarification on issues raised in the drama. Health personnel, including nutritionists from the Ghana Health Service and local radio presenters, facilitated radio discussions to throw more light on messages and issues raised in the radio drama series. The campaign was also reinforced with radio spots, which were played back 7 d/week, up to three times per day for the whole duration of the intervention.

The drama series were designed to promote the following nutrition behaviours and practices, which were identified in a formative research prior to the intervention:

Behaviour 1: Pregnant women need extra food energy and should consume foods rich in nutrients daily for healthy and strong babies.
Behaviour 2: Start feeding your child with the right food consistency only when child is 6 months of age.
Behaviour 3: Mothers of their children aged 6–24 months should feed foods that should include animal-source protein (meats, fish and eggs) and legumes daily.
Behaviour 4: Mothers of children aged 6–23 months feed nutrient-rich green leafy vegetables/fruits to their children at least three times per week.
Behaviour 5: Husbands should provide for all family members, including supporting pregnant and lactating women, to eat adequate and nutritious meals.
Behaviour 6: Prepare nutritious balanced diets using the four food groups (the four star-diet).

The intervention was implemented from January 2019 to December 2019 and consisted of the following four main activities:

(1) A formative barrier analysis was conducted to understand the influencing factors and barriers/obstacles to optimal child feeding practices. Information gathered from formative research was used by scriptwriters to translate technical nutrition messages into culturally acceptable messages using local dialects as key factors for the successful implementation of the intervention.
(2) Quantitative baseline survey to collect information about nutrition knowledge and practices among mothers.
(3) Public nutrition education on radio targeting mothers, fathers and grandmothers to improve nutrition practices.
(4) Quantitative end-line survey to evaluate changes in knowledge, attitudes, feeding practices and child growth.

The comparison communities were not exposed to nutrition messages via radio as they were separated by distance and language barrier, and this prevented the contamination of the radio messages.

### Data collection methods

The data were collected using a pre-tested and structured interviewer-administered questionnaire, which consisted of items on the respondents’ socio-demographic characteristics, anthropometric measurements of child, knowledge on IYCF, attitude towards key nutritional recommendations, dietary diversity practices and other nutritional variables. The assessments took place in the homes of participants.

The data collectors were university graduates who were fluent in the local language and received 2-d training on the study objectives, method of data collection and the tools for data collection. The data collection teams were trained and equipped to collect data electronically using smartphones and other forms of Personal Device Assistant (PDA) platforms. This process supported improved data quality and time delays inherent in the entry of data collected using paper questionnaires. Data collected each day were double-checked by the field supervisors.

### Measurement of independent and dependent variables

The primary independent variable was listening to radio health and nutrition messages (yes *v.* no) at the time of the survey. Key dependent variables of the study were (1) nutrition knowledge score (NKS), (2) attitude score, (3) nutritional status of children and (4) dietary diversity score.

### Exposure to nutrition education on radio

Participants were asked whether or not they had listened to radio drama/jingles on food and nutrition within the past 6 months, the frequency of listening to radio and to recall the messages they heard on the radio without any prompts or cues from the interviewer.

### Anthropometric assessment

The length and weight of children were taken using standardised procedures^(11,12)^. The length of the children less than 24 months was measured with an infantometer in recumbent position to the nearest 0⋅1 cm. Height was measured when subjects were standing straight with feet together, buttocks and lower back touching the wall (infantometer), subject looking straight ahead without footwear and long hair well positioned. The weight of children was taken with minimal clothing using a digital SECA 890 digital scale to the nearest 0⋅1 kg.

Anthropometric measures were converted to indices of length-for-age (LAZ), weight-for-age (WAZ) and weight-for-length (WLZ) as per the World Health Organization guidelines^(13)^.

### Assessment of nutrition-related knowledge and attitudes

The nutrition knowledge, attitudes and practices (KAP) were adapted from the FAO Knowledge Attitude and Practice Manual^(14)^. Nutrition-related knowledge was assessed as part of the structured questionnaire administered. Mothers were tested on a set of multiple-choice questions that bother on nutrition and appropriate feeding practices. The health- and nutrition-related attitudes were derived from assessing eighteen key behaviours/statements, related to appropriate child feeding, personal hygiene and uptake of health services. They were measured on a three-point Likert scale (response options ranged from ‘agree’, ‘neutral’ and ‘disagree’). A score of 0 was given to responses that disagree, 1 for being neutral and 2 for ‘agree’, yielding a summative altitude score for each respondent. A higher score meant a more positive attitude for preventive nutrition/health behaviours including appropriate IYCF practices. The scores from the items were summed up to get a total score. Scores of respondents were categorised as high if total correct responses were greater than or equal to the median score of questions related to health/nutritional attitude; otherwise, it was regarded as low.

### Assessment of CF practices

Indicators used for assessing the appropriateness of the CF practices were those described in the WHO guidelines^16^, and they included the minimum meal frequency (MMF), the minimum dietary diversity (MDD) and the minimum acceptable diet (MAD). The dietary intake of infants was determined based on the 24-h dietary recall of mothers. The seven food groups used for the determination of these indicators were (i) grains, roots and tubers; (ii) legumes and nuts; (iii) dairy products; (iv) flesh foods (meat, poultry and fish); (v) eggs; (vi) vitamin A-rich fruits and vegetables; (vii) other fruits and vegetables.

MMF is the proportion of children who received complementary foods for the minimum recommended number of times in 24 h. A child was judged to have taken ‘adequate number of meals if he/she received the minimum frequency for appropriate complementary feeding (i.e. 2 times for 6–8 months and 3 times for 9–11 months, 3 times for children aged 12–23 months) in last 24 h’. For non-breast-fed children, the MMF was 4. The WHO defined MDD as the proportion of children aged 6–23 months who received foods from at least four out of seven food groups^(15,16)^. MAD is defined as the proportion of infants aged 6–23 months who met both MMF and MDD.

### Statistical analysis

The statistical analyses were performed using the Statistical Package for the Social Sciences, version 22.0 (SPSS, Chicago, IL, USA). The analysis of data took into account the complex design of multi-stage cluster surveys. Data analysis was done using SPSS version 22.0 with the complex survey module. This was done to make statistically valid population inferences and computed standard errors from sample data. Design weights were added to each district's sample data (i.e. the total population divided by the number of respondents) to perform weighted analysis.

The nutritional indicators of child were computed using the Anthro Plus, which converted anthropometric measures into *Z*-scores. Before performing analysis with the anthropometric indices of WHZ, HAZ and WAZ, the data were cleaned, and outliers removed. The exclusion of *Z*-scores, which were outside the WHO flags: WHZ −5 to 5; HAZ −6 to 6; WAZ −6 to 5, was excluded from the data set.

The effect of health/nutrition education on radio was assessed using difference-in-difference (DID) models that assessed differences between the two study groups over time^(17)^. DID is an analytical tool used to estimate treatment effects comparing the pre- and post-treatment differences in the outcome variables of a treatment and a control group. DID is usually implemented as an interaction term between time and treatment group dummy variables in a regression model.

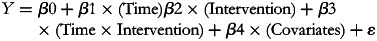
Variables with a *P* value of less than 0⋅1 in the bivariate analysis were included in the multivariable regression analysis. Multicollinearity among the predictor variables was checked before their inclusion in the final regression model. Results are presented as standardised beta coefficients with 95 % confidence interval (CI). For all analyses, the significance level was set at a *P* value of less than 0⋅05.

### Ethical considerations

The School of Allied Health Sciences of the University for Development Studies, Ghana approved the study protocol. Informed written consent was obtained from participants who could read and write. In situations, where the participants were illiterates, verbal informed consent was sought after providing the needed information and explanation. Participants were provided copies of the signed forms for their records.

## Results

### Comparison of the socio-demographic characteristics of study participants at baseline

The mean ages of the respondents in the intervention and comparison communities were similar (28⋅6 ± 6⋅7 *v.* 27⋅9 ± 5⋅7 years). Table 1 compares the details of the socio-demographic characteristics of the respondents in the study groups. The baseline characteristics of the study participants in the study groups were comparable except with respect to religion and the type of occupation. These baseline differentials were adjusted for in establishing relationships between outcomes and exposure.

**Table 1.** Comparison of the socio-demographic characteristics of study groups at baseline

		Study groups		
Variable	*N*	Comparison	Intervention	Test statistic
		*n* (%)	*n* (%)	
Age of mother (years)				
16–34	577	252 (43⋅7)	325 (56⋅3)	*χ*^2^ 1⋅0, *P* 0⋅1
≥35	135	48 (35⋅6)	87 (64⋅4)	
Maternal education				
None	469	199 (42⋅4)	270 (57⋅6)	*χ*^2^ 0⋅1, *P* 0⋅9
Low (primary and JHS)	197	82 (41⋅6)	115 (58⋅4)	
High (at least SHS)	46	19 (41⋅3)	27 (58⋅7)	
Marital status				
Not married	22	9 (40⋅9)	13 (59⋅1)	*χ*^2^ 0⋅01, *P* 0⋅9
Married	690	291 (42⋅2)	399 (57⋅8)	
Religion				
Christianity	207	7 (3⋅4)	196 (96⋅6)	*χ*^2^ 216⋅2, *P* < 0⋅001
Islam	506	293 (57⋅9)	213 (42⋅1)	
ATR	3	0 (0⋅0)	3 (100⋅0)	
Mother's occupation classification				
Trader/vendor	108	0 (0⋅0)	108 (100⋅0)	*χ^2^* 113⋅2, *P* < 0⋅001
Agricultural worker	359	177 (49⋅3)	182 (50⋅7)	
Service worker (e.g. hairdresser and seamstress)	92	55 (59⋅8)	37 (40⋅2)	
Office worker (civil servant)	36	26 (72⋅2)	10 (27⋅8)	
None	117	42 (35⋅9)	75 (64⋅1)	

### Exposure to radio listening

Radio listening characteristics of the study populations are shown in Table 2. A greater proportion of respondents listened to radio (80⋅9 %), and the popular radio stations respondents listened to included Zaa Radio and Simili Radio (Dalung), which reach out to the Savelugu and Tolon Districts. Nabiina Radio is the sole radio station in the Kassena/Nankana District in Navrongo. Radio Wa and Tumpaani FM in the Upper West Region also have a good listenership. Of those who listened to radio, 197 (60⋅4 %) listened to a radio drama/jingles on food and nutrition within the past 6 months at the end-line survey.

**Table 2.** Exposure to radio listening in intervention communities

	Baseline survey	End-line survey
Variable	*N* (%)	*N* (%)
Do you listen to radio?		
No	115 (27⋅9)	77 (19⋅1)
Yes	297 (72⋅1)	326 (80⋅9)
Which radio station do you often listen		
Cannot mention the radio	5 (1⋅7)	8 (2⋅5)
Simili Radio (Dalung)	48 (16⋅2)	61 (18⋅7)
Amaana	3 (1⋅0)	18 (5⋅5)
Filla FM	3 (1⋅0)	2 (0⋅6)
Radio Progress	46 (15⋅5)	20 (6⋅1)
Justice FM	4 (1⋅3)	3 (0⋅9)
Nabiina Radio	56 (18⋅9)	65 (19⋅9)
Radio Savannah	16 (5⋅4)	5 (1⋅5)
Radio Upper West	12 (4⋅0)	4 (1⋅2)
Radio Wa	35 (11⋅8)	30 (9⋅2)
Zaa Radio	57 (19⋅2)	59 (18⋅1)
W Fm	12 (4⋅0)	13 (4⋅0)
Tumpaani FM	0 (0⋅0)	30 (9⋅2)
Pio FM	0 (0⋅0)	8 (2⋅5)
Frequency of listening to radio		
Everyday	144 (35⋅0)	145 (36⋅0)
At least 4 d/week	62 (15⋅0)	⋅6)
Less than 4 d/week	91 (22⋅1)	90 (22⋅391 (22)
Not applicable	115 (27⋅9)	77 (19⋅1)
Time for listening to radio		
Early morning (18–21 h)	75 (18⋅2)	56 (13⋅9)
Mid-morning (22–24 h)	29 (7⋅0)	19 (4⋅7)
Afternoons (13–18 h)	46 (11⋅2)	30 (7⋅4)
After 19 h	147 (35⋅7)	221 (54⋅8)
Not applicable	115 (27⋅9)	77 (19⋅1)
Main source of nutrition and health information		
MM	125 (30⋅3)	156 (38⋅7)
Health professionals	277 (67⋅2)	233 (57⋅8)
Family and friends	10 (2⋅4)	14 (3⋅5)
Listened to a radio drama/jingles on food and nutrition within the past 6 months		
No	297 (100⋅0)	129 (39⋅6)
Yes	0 (0⋅0)	197 (60⋅4)

### Effect of nutrition education on radio on health-/nutrition-related KAP

The DID analysis was used to compare the changes over time in health- and nutrition-related KAP in intervention and control communities (Table 3). Controlling for covariates, including mother's age, mother's education, ethnicity and nutrition education on radio, was associated positively with a health-/nutrition-related KAP score as reflected in the coefficient of the interaction term (time × treatment).

**Table 3.** Effect of the intervention on health- and nutrition-related KAP (DID analysis)

Model	Unstandardised coefficients		Standardised coefficients	Sig.	95⋅0 % confidence intervals for *β*		Collinearity statistics	
	*β*	Standard errors	*β*		Lower bound	Upper bound	Tolerance	VIF
Constant	24⋅000	0⋅865		<0⋅001	22⋅30	25⋅70		
Time × treatment interaction	0⋅646	0⋅133	0⋅141	<0⋅001	0⋅39	0⋅91	0⋅986	1⋅014
Mother's age (≥35 years)	0⋅925	0⋅329	0⋅081	0⋅005	0⋅28	1⋅57	0⋅996	1⋅004
Main source of nutrition information (family and friends)	−1⋅184	0⋅268	−0⋅135	<0⋅001	−1⋅71	−0⋅66	0⋅876	1⋅141
Listen to radio	3⋅434	0⋅336	0⋅318	<0⋅001	2⋅77	4⋅09	0⋅854	1⋅172
Ethnicity of respondent (Gonja)	−1⋅103	0⋅105	−0⋅307	<0⋅001	−1⋅31	−0⋅89	0⋅954	1⋅049

To get CIs for the DID in mean *Z*-scores, OLS regression was run:

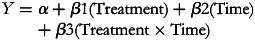


The unstandardised regression coefficient (*β*3) estimates that the DID shows the expected mean change in a KAP score for mothers in the intervention communities, which was significantly higher than their counterparts in the comparison communities (DID 0⋅646, *P* < 0⋅001).

Compared to younger women, older women (at least 35 years) had a KAP score, which was significantly higher by 0⋅925 unit. Compared to respondents who did not listen to radio, those who listened to radio had a mean KAP score, which was significantly higher by 3⋅434 units. Compared to respondents who were Dagomba by tribe, women who are Gonja and residents in the comparison district had a mean KAP score, which was significantly lower by 1⋅103 units. Women whose main source of nutrition information was family and friends had a significant health and nutrition knowledge and attitude score (*P* < 0⋅001), compared to their counterparts whose source of information was the MM.

### DID analysis comparing the changes over time in IYCF practices

After 12-month implementation of intervention activities, there were significant changes between the intervention and control arms over time with regard to some CF indicators. MDD and MAD improved significantly (DID 9⋅7 percentage points, *P* 0⋅014 and DID 12⋅1 percentage points, *P* 0⋅001, respectively) in the intervention study group, compared to the comparison group from baseline to end-line. Children in the intervention communities made greater improvement in the proportion of children meeting appropriate CF (DID 15⋅5, *P* < 0⋅001) (Table 4).

**Table 4.** Impact of radio nutrition education on child feeding practices

	Intervention communities			Comparison communities			DID
Core IYCF indicators	Baseline, *n* (%)	Follow-up, *n* (%)	Difference	Baseline, *n* (%)	Follow-up, *n* (%)	Difference	
CF rate foods at 6 months	263 (88⋅9)	228 (91⋅9)	3⋅0	237 (79⋅3)	221 (80⋅4)	1⋅1	2⋅2
MMF^a^	204 (81⋅3)	200 (93⋅0)	11⋅7	173 (57⋅7)	266 (96⋅4)	38⋅7	−27⋅0***
MDD (≥4 food groups)	75 (25⋅7)	121 (49⋅0)	23⋅3	5 (1⋅7)	42 (15⋅3)	13⋅6	9⋅7*
MAD^b^	63 (25⋅4)	106 (51⋅2)	25⋅4	5 (1⋅7)	41 (15⋅0)	13⋅3	12⋅1**
Appropriate complementary^c^	55 (22⋅4)	100 (49⋅3)	26⋅9	3 (1⋅0)	34 (12⋅4)	11⋅4	15⋅5***

a MMF is defined as two times for breast-fed infants aged 6–8 months; three times for breast-fed children aged 9–23⋅9 months; four times for non-breast-fed children aged 6–23⋅9 months. ‘Meals’ include both meals and snacks, and frequencies are based on mother's report.

b Acceptable diet is defined as who had at least the MDD and the MMF during the previous day.

c Appropriate complementary is defined as having met MAD and complementary food being introduced at 6 months.

* *P* < 0⋅05; **P < 0⋅01; ***P < 0⋅001.

After controlling for potential confounders, including educational level of mother, adequacy of meal frequency, age of child and mother's NKS, the DID analysis showed that MDD was significantly different in intervention and comparison groups (DID 9⋅316, *P* 0⋅002) (Table 5), indicating that the nutrition education impacted positively on dietary diversity for children.

**Table 5.** Factors affecting MDD (multiple linear regressions)

	Wald	Sig.	Exp(*β*)	95 % Confidence intervals for Exp(*β*)	
				Lower	Upper
Treatment (intervention)	34⋅74	<0⋅001	28⋅24	43⋅63	187⋅82
Meeting MMF	71⋅22	<0⋅001	4⋅08	2⋅94	5⋅66
Age of child (months)	75⋅19	<0⋅001	1⋅11	1⋅09	1⋅14
Mother's educational level (reference: none)	16⋅81	<0⋅001			
Low (up to JHS)	12⋅36	<0⋅001	1⋅70	1⋅27	2⋅29
High (at least SHS)	7⋅12	0⋅008	2⋅19	1⋅23	3⋅88
Time (end-line survey)	9⋅54	0⋅002	21⋅50	3⋅07	150⋅70
Knowledge score (NKS)	4⋅62	0⋅03	1⋅04	1⋅003	1⋅07
Interaction (time × treatment)	9⋅32	0⋅002	0⋅208	0⋅08	0⋅57
Constant	161⋅88	<0⋅001	0⋅006		

Children in the intervention group were twenty-eight times more likely of having minimum diet diversity at end-line compared with children in the comparison group (AOR 28⋅24; 95 % CI: 43⋅63, 187⋅82). Compared to women who had no formal education, women of highest educational level (at least senior high school) were two times (AOR 2⋅19; 95 % CI: 1⋅23, 3⋅88) more likely of providing diversified diets to their children. Compared to children who did not meet MMF, those who met MMF were four times (AOR 4⋅08; 95 % CI: 2⋅94, 5⋅66) more likely of meeting MDD. A one-unit increase in child's age was associated with 11 % higher odds of having minimum diet diversity (AOR 1⋅11, 95 % CI: 1⋅09, 1⋅14).

A one-unit increase in mother's NKS was associated with 4 % higher odds of providing minimum diet diversity (AOR 1⋅04; 95 % CI: 1⋅003, 1⋅07).

The set of variables accounted for 44⋅6 % of the variance in DDS (adjusted *R*^2^ 0⋅446).

### Impact of nutrition education on radio on nutritional status of children

Nutrition education on radio did not impact on nutritional status (Table 6). The prevalence of wasting and underweight was significantly higher in the intervention districts, but there was no significant difference in stunting.

**Table 6.** Comparison of changes in nutritional indicators in the intervention and comparison communities

	Intervention communities			Comparison communities			DID
Indicators	Baseline	Follow-up	Difference	Baseline	Follow-up	Difference	
Mean HAZ	−0⋅98	−1⋅11	−0⋅13	−1⋅16	−0⋅73	0⋅43	−0⋅55**
Mean WAZ	−0⋅92	−1⋅12	−0⋅2	−1⋅26	−0⋅79	0⋅47	−0⋅67***
Mean WHZ	−0⋅57	−0⋅74	−0⋅17	−0⋅85	−0⋅30	0⋅55	−0⋅72***
Stunted (HAZ < −2), *n* (%)	101 (25⋅1)	108 (26⋅9)	1⋅8	85 (28⋅4)	72 (24⋅4)	−4⋅0	5⋅8
Wasted (WHZ < −2), *n* (%)	43 (10⋅6)	58 (14⋅4)	3⋅8	55 (18⋅3)	18 (6⋅6)	−11⋅7	15⋅5***
Underweight (WAZ < −2), *n* (%)	76 (18⋅5)	93 (23⋅1)	4⋅6	77 (25⋅7)	38 (13⋅9)	−11⋅8	16⋅4***

**P < 0⋅01; ***P < 0⋅001.

## Discussion

This intervention study evaluated whether nutrition communication strategy using innovative drama series on radio is associated with improved nutritional knowledge of mothers, positive preventive health-seeking behaviours and practices such as feeding diversified foods to children. Our analysis provided evidence that health and nutrition education using radio drama significantly and positively increased health-/nutrition-related KAP but had little effect on the nutritional status of children.

### Effect of nutrition education on mothers’ nutrition knowledge, attitude and child feeding practices

In this study, the intervention group had shown statistically significant improvement in KAP of the mothers/caregivers compared to the control group.

The results of this study are therefore consistent with similar behaviour change interventions carried out elsewhere where nutrition behaviour change communication impacted positively on MDD and MAD^(18–21)^.

A comparative study of Radio Listening Club (RLC) members and non-RLC members in Malawi showed a statistically significant difference between RLC members and non-RLC members in relation to KAP regarding IYCF^(22)^.

Recent nutrition education interventions in Ethiopia were found effective in improving mother/caregiver behaviours related to knowledge and child feeding practices^(23–25)^. The effectiveness of nutrition education on improving knowledge and practice of CF has also been reported from Kenya, China and Uganda^(3,20,26)^

### Effect of nutrition education on nutritional status of children

The nutrition education on radio did not have significant effects on the nutritional status as measured by height-for-age (HAZ) *Z*-score or weight-for-height (WHZ) *Z*-score. This is because the prevalence of wasting and underweight was significantly higher in the intervention districts, but there was no significant difference in stunting.

The increased prevalence of wasting and underweight in the intervention district is different from what one would expect in most nutrition interventions. Noteworthy is the fact that this happened amidst improvement in mothers’ nutrition-related knowledge and practices.

It is unclear what might have contributed to the deterioration in wasting and underweight of children aged 6–36 months but may be due to unfavourable environmental and socio-economic changes, which the ongoing nutrition intervention could not have addressed. Malnutrition in children occurs because of the interplay of various factors such as socio-demographic, maternal, gender, home environment, dietary practices, hand washing and other hygiene practices.

Wasting as an indicator of acute malnutrition manifests as a consequence of insufficient food intake or a high incidence of infectious diseases, especially diarrhoea. Changes in acute malnutrition can occur very rapidly within short periods. Underweight is a composite indicator of wasting and stunting, so any changes in wasting will affect underweight.

Furthermore, a decrease in wasting and underweight was observed in the control cohort following the 1-year study period. The situation may partly be explained by the fact that during our intervention period, UNICEF implemented a pilot project in the comparison district which aimed to prevent stunting and micronutrient deficiencies among children aged 6–23 months by promoting both the consumption of locally available nutritious foods and lipid-based nutrient supplement – small quantity using social and behaviour change communication (SBCC) strategies. This might have confounded our results.

It is worthy to note, however, that similar other nutrition education interventions have not been able to positively affect the linear growth of children in many other settings including South Africa, Bangladesh, Brazil and India^(27–30)^. Adequate knowledge *per se* is not always translated into appropriate actions as evidenced in several studies^(31,32)^. In Ethiopia, nutrition education that promoted IYCF messages significantly improved KAP scores regarding CF, as well as dietary diversity, but there were no changes in height and weight between the study groups^(33)^. Similarly, another intervention that combined interpersonal communication and a national MM campaign with either intensive or non-intensive community mobilisation (CM) made greater improvements in dietary diversity in the group with intensive CM, though changes in child growth were not observed^(34)^.

The lack of linear growth observed in this and other studies may be due to either the short intervention period or the advanced age of the targeted children. Usually, greater improvements in growth are found when younger children are targeted before stunting is already prevalent^(35)^. The relatively short intervention period could not have allowed enough time for significant linear growth to occur.

The nutritional status of an individual is influenced by a lot of interrelated and complex factors at both household and community levels. At the household, nutritional status is affected by the household ability to provide adequate food in both quantity and quality, care from caretakers/mothers and nutrition knowledge, especially that of the mother and other socio-cultural factors. With an improved nutritional knowledge of mothers, it is expected that they will have better feeding practices for their children, thereby preventing the risk of malnutrition. It is undeniable fact that mothers with the requisite nutritional knowledge together with other equally important resources will be able to adequately care for the children to grow and develop optimally. However, a mother who is deprived of economic resources may not provide adequate care no matter the wealth of knowledge she has. Therefore, nutrition education *per se* is insufficient to have any positive effect on child growth if the food being promoted is not available in the first place. It is to be noted that the success of nutrition education interventions could be influenced by some other factors that are not accounted for in most studies. In particular, it is a known fact that low-resource households may not simply change their behaviour if food is unavailable, so strategies must combine behaviour change with food access. In view of the complexities involved in behaviour change, it would not be enough to simply educate or give advice to people and expect that change will automatically take place. This is because knowing something is ‘good for you’ is rarely sufficient to change behaviour. According to Kwasnicka *et al.*^(36)^, successful behaviour change requires the target population to (i) engage with the need to change, (ii) sustain the motivation to maintain the change and (iii) be supported by contexts (service providers, society, social networks and environments) that facilitate change.

Effective utilisation of knowledge and skills gained from health and nutrition education is expected to improve the health and nutritional status of children through improved knowledge and care practices. However, some studies have reported that maternal nutritional knowledge is positively associated with the nutritional status of children^(37–39)^.

## Conclusion

Raising awareness of nutrition and preventable health-related issues through radio messaging increased maternal nutritional knowledge and child's dietary diversity, which may contribute significantly to child growth if there is concurrent improvement in other barriers such as food insecurity and socio-economic constraints of households.

### Policy implications

The study findings highlight the potential of using MM in the form of radio drama to increase health-/nutrition-related knowledge, practices and behaviours but underscore the reality that nutrition education alone may not be sufficient to positively impact on nutrition. Nutrition communication campaigns may be implemented in conjunction with other interventions that can leverage the individual from knowledge to practice.

### Study limitations

Although a quasi-experimental design was used to evaluate the intervention, there are some limitations worth mentioning. Firstly, the design has an inability to sufficiently control all important confounding variables arising from the lack of randomisation. This meant not all alternative explanations could be accounted for, so statistical association identified did not imply causality. Secondly, recall bias cannot be ruled out, as dietary data were collected retrospectively; study participants had to recall before they could respond to some questions.

Another significant limitation of this study is the implementation of another nutrition intervention in the comparison district during our study period by UNICEF.

## Supplementary material

For supplementary material accompanying this paper visit https://doi.org/ 10.1017/jns.2021.35. click here to view supplementary material
